# Changes in Temporomandibular Disorder Symptoms Following Occlusal Splint Therapy: A Prospective Single-Arm Before-After Study

**DOI:** 10.7759/cureus.116299

**Published:** 2026-09-15

**Authors:** Juan Antonio Maldonado-Moreno, Luis Pablo Cruz-Hervert, Martha Elizabeth Tovar-Martínez, Álvaro Edgar González-Aragón Pineda, Gustavo Reza-Bravo, Jesus Alberto Rocha Ortíz, Cecilia Carlota Barrera-Ortega, Gerardo Martínez-Suárez

**Affiliations:** 1 Department of Surgery and Orthodontics, Regional High Specialty Hospital of Ixtapaluca, Ixtapaluca, MEX; 2 Division of Graduate Studies and Research, Faculty of Dentistry, National Autonomous University of Mexico (UNAM), Coyoacán, MEX; 3 Faculty of Higher Studies Iztacala, National Autonomous University of Mexico (UNAM), Tlalnepantla, MEX; 4 School of Public Health, Autonomous University of Chihuahua, Chihuahua, MEX; 5 Faculty of Higher Studies Zaragoza, National Autonomous University of Mexico (UNAM), Iztapalapa, MEX

**Keywords:** bite splint, occlusal splint, temporomandibular disorders, temporomandibular dysfunction, tmj pain

## Abstract

Objective

The objective of this study was to evaluate changes in symptoms associated with temporomandibular disorders (TMDs) before and after occlusal splint therapy.

Materials and methods

We conducted a prospective, single-arm, uncontrolled before-after intervention study including 76 patients aged 14-60 years who attended the Orthodontics Department at the Regional High Specialty Hospital of Ixtapaluca, Ixtapaluca, State of Mexico, between March and December 2024. All participants were diagnosed with TMD according to the International Classification of Diseases, Tenth Revision (ICD-10). Patients presenting with temporomandibular joint or masticatory muscle pain and/or difficulty opening or closing the mouth were included, whereas those with degenerative joint disease, condylar fractures, or complete edentulism were excluded. Dental casts and clinical records were obtained, and a symptom questionnaire was administered before and after occlusal splint therapy. Data were analyzed using the chi-square and McNemar tests.

Results

A total of 76 patients were included, comprising 85.5% (n = 65) women and 14.5% (n = 11) men. At baseline, 100.0% (n = 76) of the participants presented at least one TMD-related symptom. The most prevalent symptom was joint clicking, reported by 69.7% (n = 53) of the participants. Muscle pain was significantly more frequent among women, affecting 47.7% (n = 31 of 65) compared with 9.1% (n = 1 of 11) of men (P = 0.020). After occlusal splint therapy, the proportion of patients presenting at least one symptom decreased from 100.0% (n = 76) to 78.9% (n = 60), whereas 21.1% (n = 16) became symptom-free (P < 0.001). Significant reductions were observed in headache, facial, or neck pain, from 48.7% (n = 37) to 14.5% (n = 11); joint fatigue, from 50.0% (n = 38) to 11.8% (n = 9); muscle pain, from 42.1% (n = 32) to 10.5% (n = 8); and joint clicking, from 69.7% (n = 53) to 40.8% (n = 31) (all P < 0.001).

Conclusions

Reductions in TMD-related symptoms were observed between baseline and the end of the six-month occlusal splint treatment period, particularly for joint fatigue, muscle pain, and headache. Given the uncontrolled before-after design, these findings should be interpreted as temporal associations and do not establish treatment efficacy. Controlled studies using standardized diagnostic criteria and validated outcome measures are needed to confirm these findings.

## Introduction

Temporomandibular disorders (TMDs) comprise a heterogeneous group of conditions affecting the temporomandibular joints (TMJs), masticatory muscles, and associated structures, often causing pain and functional impairment that compromise normal mandibular function [1-3]. Clinically, TMDs present symptoms such as joint and muscle pain, headaches, joint sounds, tinnitus, and limited mandibular movement. According to their etiology, TMDs may be classified as myogenic, arthrogenic, or mixed, with pain of somatic or neurogenic origin [4,5]. Epidemiological studies have reported varying frequencies of TMD-related signs and symptoms across different populations and according to the assessment methods used [6].

Risk indicators and associated factors for TMD may include skeletal discrepancies, malocclusion, parafunctional habits, psychological stress, trauma, hormonal influences, nutritional deficiencies, and previous craniofacial surgery [7,8]. Psychological factors have shown a strong association with TMDs. Marpaung et al. [8] reported an association between anxiety and pain and joint noises, while Restrepo et al. [9] reported associations of stress and related biomarkers with myofascial pain and TMD-associated headaches, even in pediatric patients.

Occlusal splints are among the most commonly used therapeutic approaches for TMD management [10-12]. Their primary purpose is to redistribute muscular forces, protect the stomatognathic system, and improve mandibular guidance. Occlusal splints may reduce pain and improve mandibular movement in some patients; however, their superiority over other conservative treatments remains uncertain, and arthrogenic and myogenic TMDs may respond similarly to treatment [13-19]. Conservative management of TMD may also include physiotherapy, self-care, behavioral therapy, pharmacotherapy, orthodontics, and surgery.

Despite their widespread use, uncertainty remains regarding the mechanisms of action of occlusal splints and the magnitude of symptom changes associated with their use, supporting further clinical evaluation of changes in TMD-related symptoms during splint therapy. Splint design and mandibular-position protocols vary considerably, and current evidence does not clearly establish the clinical superiority of fabrication based on centric relation over alternative mandibular positions [20-22]. Recent evidence, including the meta-analysis by Zhang et al. [11], demonstrated that occlusal splints significantly reduce pain and improve mouth opening in patients with restricted mandibular function. TMDs affect approximately 31.1% of the global population and up to 66% in Mexico [23-25], with a higher prevalence in women, possibly related to hormonal and psychosocial factors [26]. Most patients improve with noninvasive therapies, including occlusal devices [27]. Therefore, this prospective, single-arm, uncontrolled before-after study evaluated patients with TMD undergoing occlusal splint therapy over a six-month period. The objective was to assess changes in patient-reported TMD-related symptoms from baseline to the end of treatment.

## Materials and methods

A prospective, single-arm, uncontrolled before-after intervention study was conducted in the Orthodontics Department between March and December 2024. This research study was conducted in accordance with the ethical principles established in the Declaration of Helsinki and was approved by the Ethics Committee. All participants provided written informed consent prior to inclusion in the study, authorizing the use of their clinical records for research purposes. For participants younger than 18 years, written informed consent was obtained from their parents or legal guardians, in addition to the participant's assent. All procedures, including the assessment of centric relationships, were performed following established clinical guidelines. The study ensured strict adherence to ethical standards, protecting patient confidentiality and rights throughout the research process. Consent also authorized the use of anonymized data for scientific publication purposes (approval NR-017-2024).

The orthodontics and maxillofacial surgery service receives about 200 new cases per year, of which 80 patients require the management of TMDs. This includes a review by an orthodontist, who must continuously implement preventive and therapeutic measures for joint complications, with joint pain being a frequent reason for consultation in these patients.

The study population consisted of patients with a clinical diagnosis of TMD who were treated. These patients were primarily referred by the maxillofacial surgery and otorhinolaryngology services. TMD diagnoses were recorded according to the International Classification of Diseases, Tenth Revision (ICD-10), during the period from March to December 2024. No formal standardized diagnostic protocol, such as the Diagnostic Criteria for Temporomandibular Disorders (DC/TMD) or Research Diagnostic Criteria for TMD (RDC/TMD), was used.

The study population consisted of male and female patients aged 14 to 60 years who attended the Orthodontics Department of the Regional High Specialty Hospital of Ixtapaluca, Ixtapaluca, State of Mexico. Participants were selected according to the inclusion and exclusion criteria summarized in Table 1. Eligibility required the presence of one or more of the listed inclusion criteria; the simultaneous presence of all clinical features was not required.

**Table 1:** Inclusion and exclusion criteria Mandibular range of motion was assessed in millimeters using a Range of Motion Scale (CPT-9585; Great Lakes Orthodontics, New York, NY, USA).

Inclusion Criteria	Exclusion Criteria
Patients aged between 14 and 60 years	Current botulinum toxin therapy for temporomandibular disorders
Presence of mandibular function alterations	Current hyaluronic acid injection therapy for temporomandibular disorders
Presence of mandibular deviation during mouth opening	Current temporomandibular joint viscosupplementation treatment
Presence of joint clicking	Degenerative joint disease affecting the temporomandibular joint
Presence of pain localized to the temporomandibular joint region, as reported by the patient during clinical assessment	Arthritis affecting the temporomandibular joint
Presence of pain in the masticatory muscles, as reported by the patient during clinical assessment	History of condylar fracture
Patient-reported functional difficulty or limitation in opening the mouth	Total edentulism
Patient-reported functional difficulty or limitation in closing the mouth	-

Mandibular range of motion assessment included maximum mouth opening, right and left lateral excursions, and protrusion. Maximum mouth opening was defined as the vertical interincisal distance at maximal voluntary opening. Lateral excursions were measured as the horizontal displacement of the mandibular midline toward each side, and protrusion as the horizontal anterior displacement of the mandible relative to the maxillary incisors. All measurements were recorded in millimeters. Mandibular deviation during opening and closing was assessed using brief video recordings and was recorded according to direction as right or left. The magnitude of deviation and the specific phase of opening in which it occurred were not quantified. Joint clicking was assessed bilaterally by palpation of the TMJ during repeated mandibular opening and closing movements. A click was considered present when a reproducible palpable joint sound was detected during mandibular movement.

Potential eligibility and exclusion factors were assessed based on the clinical records available at the time of patient selection. The study did not prospectively collect standardized information on all potential confounders related to previous treatments, medication use, behavioral factors, or systemic conditions.

No a priori sample size calculation was performed. All eligible participants available during the study period who met the predefined inclusion and exclusion criteria were included in the study, resulting in a final sample of 76 participants.

A total of 76 patients received treatment for the signs and symptoms of TMD using an occlusal splint over a period of six months, divided into three phases: initial (0-2 months), intermediate (2-4 months), and final (4-6 months). The evaluation of signs and symptoms was conducted at the beginning and end of treatment using a symptom questionnaire administered before and after occlusal splint therapy. This questionnaire allowed for the identification of symptoms associated with TMD, such as dizziness, headaches, reduced hearing or tinnitus (ringing in the ears), joint noises, fatigue when chewing, neck pain, limitation in mouth opening or closing, and bruxism. Pain intensity reported by each patient was assessed using a visual analog scale, while the TMD-related symptom items were recorded as present or absent.

The questionnaire was non-validated and was administered at both assessment time points using the same format. Symptom items were recorded as present or absent, while pain intensity was recorded using a visual analog scale. The questionnaire assessed symptom presence rather than frequency or functional interference. No formal language validation or translation procedure was performed, and the assessment was not blinded to the time point.

Significant symptomatology was considered when the participant reported three or more symptoms, following operational criteria due to the variability of diagnostic cut-off points in TMD reported in the literature [28-30]. At the six-month follow-up evaluation, participants were asked about their perceived effectiveness of the occlusal splint and their willingness to recommend its use to others. Self-perception of the effectiveness of the occlusal splint was assessed using a four-category Likert-type response scale: strongly agree, somewhat agree, neither agree nor disagree, and strongly disagree. For statistical analysis, responses were dichotomized into a positive perception (“Yes”: strongly agree or somewhat agree) and a non-positive perception (“No”: neither agree nor disagree or strongly disagree). Sociodemographic data such as age, sex, age group, and duration of occlusal splint use were also collected. Participants were also classified according to the stage of study recruitment at which they entered the study as initial (n = 18), intermediate (n = 16), or final (n = 42). This classification reflected the recruitment period only and did not represent differences in treatment duration or follow-up; all participants underwent the same six-month treatment protocol.

The evaluation of the signs and symptoms associated with TMD was carried out only in the first and last sessions. Among the recorded symptoms were patient-reported functional limitation in mouth opening or closing, pain during mouth opening or closing, joint pain, and masticatory muscle pain, as well as the presence of audible noises in the TMJs and other symptoms described above. At each monthly appointment, the corresponding adjustment to the occlusal splint was also made under the protocol of point contacts, bilateral and simultaneous. No concomitant TMD-specific treatments, including analgesic medication, physiotherapy, psychological treatment, or other therapeutic interventions, were prescribed as part of the study protocol during the six-month treatment period.

Patients were followed through monthly appointments during the six-month treatment period, during which the occlusal splint was adjusted as required. Duration of splint use was recorded; however, a standardized daily wearing schedule and a predefined adherence threshold were not prospectively documented.

Neuromuscular deprogramming was performed using a Lucia Jig (AD2®, Advanced Dental Designs, Inc., Moreno Valley, CA, USA) for 10 minutes [31]. Centric relation was subsequently recorded using DeLar Blue Bite Registration Wax (DeLar Corp., Lake Oswego, OR, USA). The wax segments were carefully adapted and cooled to minimize deformation and were then used as a guide to position the mandible in centric relation while maintaining a 1.5-mm posterior occlusal separation. The casts were mounted using a Panadent articulator, facebow, mounting kit, bite tabs, and mounting table (Panadent Corporation, Colton, CA, USA).

Although maxillomandibular records using the centric power technique of Roth may present technical difficulties, the clinicians participating in this study were trained to perform the procedure uniformly [32]. The same fabrication and adjustment protocol was applied throughout the study, and the splint was subsequently adjusted at monthly appointments according to the protocol of bilateral and simultaneous point contacts. For the fabrication of the occlusal splint in the upper arch, plaster was applied to areas with potential retention, followed by precise wax adaptation. Before the flasking process, the occlusal contacts and functional guides were checked, which allowed for the final occlusal splint to be obtained (Figure 1).

**Figure 1:**
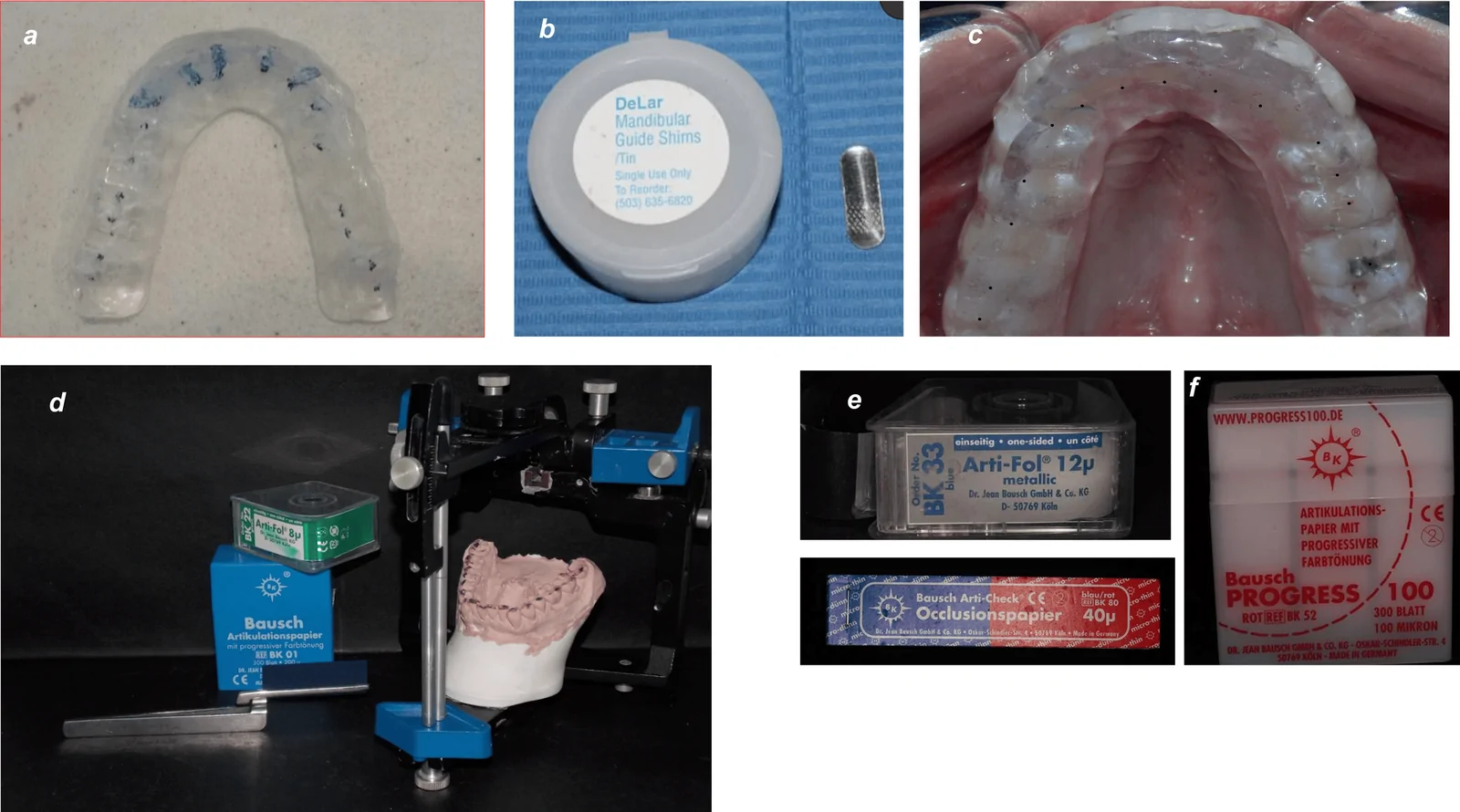
Occlusal splint therapy and materials used for mandibular repositioning Materials and procedures used for occlusal splint adjustment and occlusal analysis. (a) Maxillary occlusal splint with reference markings. (b) Maxillary guide shims used for occlusal calibration. (c) Intraoral view of the maxillary occlusal splint in place showing marked occlusal contacts. (d) Dental casts mounted on a semi-adjustable articulator for occlusal evaluation. (e) The articulating papers used to identify and verify occlusal contacts were, from thickest to thinnest: Bausch Arti-Check® Occlusion Paper, 40 μm (Ref. BK 80), and Bausch Arti-Fol® Metallic, 12 μm (Dr. Jean Bausch GmbH & Co. KG, Köln, Germany). Different paper thicknesses were used to facilitate both the initial identification and the precise verification of occlusal contacts. (f) During occlusal splint adjustment, Bausch Progress® 100, 100 μm (Ref. BK 52; Dr. Jean Bausch GmbH & Co. KG, Köln, Germany) was used to identify and verify occlusal contacts during the adjustment procedure.

Participants were classified into three age groups for analysis: 14-29, 30-59, and ≥60 years. Age eligibility was determined at baseline. Participants aged 60 years at enrollment were retained in the ≥60-year category for analysis even if they turned 61 before the six-month follow-up.

The data analysis was conducted using Stata 14 software (Stata Corp., College Station, TX, USA). Descriptive statistics were calculated, and chi-square and Fisher's exact tests were applied to evaluate the differences in symptom prevalence between categorical variables. To analyze changes in paired binary symptom variables before and after treatment, the McNemar test was used. A statistical significance level of p < 0.05 was considered. No primary outcome was prospectively specified, and no adjustment for multiple comparisons was applied; therefore, analyses of individual symptoms should be interpreted as exploratory.

## Results

The study included 76 participants, of whom 85.5% (n=65) were women and 14.5% (n=11) were men, with a mean age of 42 ± 15.9 years. All 76 participants completed both the baseline and six-month follow-up assessments, with no losses to follow-up or missing data in the variables included in the analyses. Age group I (14-29 years) comprised 19 (25.0%) participants with a mean age of 18 ± 3.5 years; group II (30-59 years) included 45 (59.2%) participants; and group III (≥60 years) comprised 12 (15.8%) participants. The most frequent symptom was joint clicking, reported by 53 participants (69.7%), followed by joint fatigue in 38 (50.0%) participants and headache, facial, or neck pain in 37 (48.7%) participants. Muscle pain was more frequently reported among women than men at baseline (47.7% [n = 31/65] vs. 9.1% [n = 1/11], respectively; Fisher’s exact test, p = 0.020) (Table 2).

**Table 2:** Symptoms related to temporomandibular disorders before treatment, categorized by sex (n=76) Statistical significance of Fisher's exact test: *p ≤ 0.05

Symptoms	Male, n (%)	Female, n (%)	Total, n (%)	χ² (df)	Fisher's exact test, P-value	Effect size
Skull facial trauma						
Absence	10 (90.9%)	61 (93.8%)	71 (93.4%)	-	-	Phi = 0.041
Presence	1(9.1%)	4 (6.2%)	5 (6.6%)	0.132 (1)	0.553	
Head, face, or neck pain						
Absence	7 (63.6%)	32 (49.2%)	39 (51.3%)	-	-	Phi = 0.101
Presence	4 (36.4%)	33 (50.8%)	37 (48.7%)	0.781(1)	0.518	
Dizziness						
Absence	9 (81.8%)	47 (72.3%)	56 (73.7%)	-	-	Phi = 0.076
Presence	2 (18.2%)	18 (27.7%)	20 (26.3%)	0.438(1)	0.717	
Reduced hearing or tinnitus						
Absence	9 (81.8%)	37 (56.9%)	46 (60.5%)	-	-	Phi = 0.179
Presence	2 (18.2%)	28 (43.1%)	30 (39.5%)	2.440(1)	0.184	
Joint clicking						
Absence	5 (45.5%)	18 (27.7%)	23 (30.3%)	-	-	Phi = 0.136
Presence	6 (54.5%)	47 (72.3%)	53 (69.7%)	1.406(1)	0.292	
Fatigue						
Absence	5 (45.5%)	33 (50.8%)	38 (50.0%)	-	-	Phi = 0.037
Presence	6 (54.5%)	32 (49.2%)	38 (50.0%)	0.106(1)	1.000	
Muscle pain						
Absence	10 (90.9%)	34 (52.3%)	44 (57.9%)	-	-	Phi = 0.275
Presence	1 (9.1%)	31 (47.7%)	32 (42.1%)	5.750(1)	0.020*	
Difficulty opening the mouth						
Absence	9 (81.8%)	51 (78.5%)	60 (78.9%)	-	-	Phi = 0.029
Presence	2 (18.2%)	14 (21.5%)	16 (21.1%)	0.063(1)	1.000	
Difficulty closing the mouth						
Absence	8 (72.7%)	56 (86.1%)	64 (84.2%)	-	-	Phi = 0.129
Presence	3 (27.3%)	9 (13.9%)	12 (15.8%)	1.275(1)	0.365	
Bruxism (teeth grinding)						
Absence	5 (45.5%)	50 (76.9%)	55 (72.4%)	-	-	Phi = 0.247
Presence	6 (54.5%)	15 (23.1%)	21 (27.6%)	4.659(1)	0.062	

The proportion of participants with TMD symptoms decreased from 100.0% (n = 76) at baseline assessment to 78.9% (n = 60) at follow-up. Consequently, 21.1% (n = 16) of the participants were symptom-free after the intervention. This reduction was statistically significant (P ≤ 0.001).

Likewise, significant reductions were observed in the proportion of participants reporting the evaluated symptoms. The proportion of participants reporting headache, facial, or neck pain decreased from 48.7% (n = 37) to 14.5% (n = 11) (P ≤ 0.001); dizziness, from 26.3% (n = 20) to 4.0% (n = 3) (P ≤ 0.001); reduced hearing or tinnitus, from 39.5% (n = 30) to 18.4% (n = 14) (P ≤ 0.001); joint clicking, from 69.7% (n = 53) to 40.8% (n = 31) (P ≤ 0.001); joint fatigue, from 50.0% (n = 38) to 11.8% (n = 9) (P ≤ 0.001); muscle pain, from 42.1% (n = 32) to 10.5% (n = 8) (P ≤ 0.001); difficulty opening the mouth, from 21.1% (n = 16) to 5.3% (n = 4) (P ≤ 0.05); difficulty closing the mouth, from 15.8% (n = 12) to 1.3% (n = 1) (P ≤ 0.001); and bruxism, from 27.6% (n = 21) to 6.6% (n = 5) (P ≤ 0.001).

Joint fatigue showed the largest decrease in the number of participants reporting the symptom, from 38 (50.0%) at baseline to 9 (11.8%) at six months (Table 3). A significant reduction in TMD symptoms after treatment was observed among women, with the proportion of symptomatic participants decreasing from 100.0% (n = 65) before treatment to 83.0% (n = 54) after treatment (P = 0.001). Significant reductions were also observed among participants aged 14-29 years, from 94.7% (n = 18) to 63.2% (n = 12) (P = 0.031), and among those aged 30-59 years, from 100.0% (n = 45) to 82.2% (n = 37) (P = 0.008).

**Table 3:** Symptoms related to temporomandibular disorders before and after treatment (n=76) †McNemar test

Symptom	Before treatment		After treatment		P-value
	Absence, n (%)	Presence, n (%)	Absence, n (%)	Presence, n (%)	
Reduced hearing or tinnitus	46 (60.5%)	30 (39.5%)	62 (81.6%)	14 (18.4%)	<0.001†
Head, face, or neck pain	39 (51.3%)	37 (48.7%)	65 (85.5%)	11 (14.5%)	<0.001†
Dizziness	56 (73.7%)	20 (26.3%)	73 (96.0%)	3 (4.0%)	<0.001†
Joint clicking	23 (30.3%)	53 (69.7%)	45 (59.2%)	31(40.8%)	<0.001†
Joint fatigue	38 (50.0%)	38 (50.0%)	67 (88.2%)	9 (11.8%)	<0.001†
Muscle pain	44 (57.9%)	32 (42.1%)	68 (89.5%)	8 (10.5%)	<0.001†
Difficulty opening mouth	60 (78.9%)	16 (21.1%)	72 (94.7%)	4 (5.3%)	0.0042†
Difficulty closing mouth	64 (84.2%)	12 (15.8%)	75 (98.7%)	1 (1.3%)	0.0010†
Bruxism (teeth grinding)	55 (72.4%)	21 (27.6%)	71 (93.4%)	5 (6.6%)	0.0001†

According to the study recruitment stage, a significant reduction in TMD symptoms was observed among participants in the final recruitment stage, with the proportion of symptomatic participants decreasing from 97.6% (n = 41) before treatment to 76.2% (n = 32) after treatment (P = 0.002).

Similarly, among participants who reported that they would recommend the use of the occlusal splint, the proportion presenting TMD symptoms decreased from 98.6% (n = 72) before treatment to 79.5% (n = 58) after treatment (P < 0.001) (Table 4).

**Table 4:** Symptoms related to temporomandibular disorders before and after treatment, by sex, age, study recruitment stage, and self-perception variables (n=76) †McNemar test

Variable	Before treatment		After treatment		Total, n (%)	P-value
	Absence, n (%)	Presence, n (%)	Absence, n (%)	Presence, n (%)		
Sex						
Male	0 (0%)	11 (100%)	5 (45.5%)	6 (54.5%)	11 (100%)	0.1250†
Female	0 (0%)	65 (100%)	11 (17.0%)	54 (83.0%)	65 (100%)	0.0010†
Age						
14 to 29 years	1 (5.3%)	18 (94.7%)	7 (36.8%)	12 (63.2%)	19 (100%)	0.0313†
30 to 59 years	0 (0%)	45 (100%)	8 (17.8%)	37 (82.2%)	45 (100%)	0.0078†
≥60 years	0 (0%)	12 (100%)	1 (8.3%)	11 (91.7%)	12 (100%)	1.0000†
Study recruitment stage						
Start	0 (0%)	18 (100%)	2 (11.1%)	16 (88.9%)	18 (100%)	0.5000†
Intermediate	0 (0%)	16 (100%)	4 (25.0%)	12 (75.0%)	16 (100%)	0.1250†
Final	1 (2.4%)	41 (97.6%)	10 (23.8%)	32 (76.2%)	42 (100%)	0.0020†
Self-perception about recommending the occlusal guard						
No	0 (0%)	3 (100%)	1 (33.3%)	2 (66.7%)	3 (100%)	1.0000†
Yes	1 (1.4%)	72 (98.6%)	15 (20.5%)	58 (79.5%)	73 (100%)	0.0001†

Table 5 shows lower post-treatment TMD-related symptom prevalence across all evaluated subgroups, as reflected by negative percentage-point differences (Δ) and prevalence ratios (PR) below 1. The largest descriptive changes were observed among men (Δ = −36.4 percentage points; PR = 0.60), participants aged 14-29 years (Δ = −31.5 percentage points; PR = 0.67), and individuals who would not recommend the appliance (Δ = −33.3 percentage points; PR = 0.67). Lower post-treatment symptom prevalence was also observed among women, participants aged 30-59 years, those in the final recruitment stage, participants who perceived the occlusal splint as effective, and those who would recommend its use. These subgroup findings should be interpreted descriptively, particularly for categories with small sample sizes.

**Table 5:** Changes in presence (%) and prevalence ratio by study variables Changes were summarized using the prevalence ratio (PR) and the difference in prevalence (Δ), expressed in percentage points (Post−Pre). Abbreviations: Pre, pre-intervention; Post, post-intervention; Δ (%), absolute percentage change (Post−Pre); PR, prevalence ratio (Post/Pre). Note: Values are expressed as percentages. The prevalence ratio (PR) reflects the relative change in presence after the intervention compared with baseline; values <1 indicate a reduction, whereas values >1 indicate an increase.

Participant subgroup		Pre-treatment (%)	Post-treatment (%)	Δ (%)	PR (post/pre)	Trend
Sex	Male	90.9	54.5	-36.4	0.60	↓
	Female	100.0	83.0	-17.0	0.83	↓
Age	14–29	94.7	63.2	-31.5	0.67	↓
	30–59	100.0	82.2	-17.8	0.82	↓
	≥60	100.0	91.7	-8.3	0.92	↓
Study recruitment stage	Initial	100.0	88.9	-11.1	0.89	↓
	Intermediate	100.0	75.0	-25.0	0.75	↓
	Final	97.6	76.2	-21.4	0.78	↓
Recommendation of the occlusal splint	No	100.0	66.7	-33.3	0.67	↓
	Yes	98.6	79.5	-19.1	0.81	↓

## Discussion

The results of the present study show that joint fatigue was the symptom that exhibited the greatest decrease after the intervention, followed by muscle pain, headache, and facial or cervical pain. The observed reductions are consistent with symptom improvement during the treatment period; however, the uncontrolled design does not permit attribution of these changes specifically to the occlusal splint. Occlusal splints constitute a conservative and non-invasive therapeutic alternative capable of improving mandibular mobility and reducing pain intensity in patients with TMD [2-8,13,14,33]. In accordance with these reports, the present study observed a consistent reduction in symptoms across all analyzed groups, with PRs less than 1 (0.60-0.92) and negative absolute changes, indicating a lower proportion of participants reporting symptoms at the post-treatment assessment compared with baseline.

However, the magnitude of the observed reductions varied across the analyzed subgroups, with greater reductions observed in men, younger patients, and those in the final recruitment stage, while statistically significant changes were mainly observed in women, those in the age group of 30-59 years, and participants with a positive perception of therapeutic efficacy.

These findings indicate that the magnitude of the observed symptom reductions varied across demographic and clinical subgroups, which is consistent with the variability reported in the literature.

Other patient-reported symptoms, including joint clicking, tinnitus, limited mouth opening, and bruxism, also showed reductions after treatment. However, these manifestations are not specific to TMD and may have different underlying causes. Therefore, their observed changes should be interpreted as symptom-level findings rather than as evidence of improvement in specific TMD diagnoses.

Regarding the frequency of symptoms, joint clicking was the most common (69.7%), followed by joint fatigue (50.0%), and headache, facial, or cervical pain (48.7%), coinciding with other studies [34,35], which reported a high prevalence of facial pain, joint noises, and headache, especially in individuals aged 18 to 20 years. Regarding sex differences, the present study found a higher prevalence of muscle pain in women compared to men, which is consistent with previous epidemiological research indicating that TMDs more frequently affect the female sex. For example, a study in Västerbotten, in northern Sweden, reported a general prevalence of 9.2%, with significantly higher rates in women 12.9% than in men (5.4%) [24,35]. These differences could be related to hormonal factors, behavioral factors, and variations in pain perception and modulation, aspects that could influence the clinical expression of TMD and the response to interventions such as occlusal splints.

Likewise, a decrease in the prevalence of symptoms was observed after occlusal splint therapy, especially in adults aged 30 to 59 years, compared to younger participants. Previous studies have reported TMD-related symptoms in different populations, although differences in study characteristics limit direct comparisons [36]. In the present study, the proportion of participants reporting at least one TMD-related symptom decreased from 100.0% at baseline to 78.9% at the six-month assessment. These findings are consistent with previous reports of reductions in TMD-related symptoms following occlusal splint therapy, although differences in study populations and outcome assessment should be considered when making comparisons [11]. Likewise, studies in the hospital population of Mexico City have reported a prevalence of TMD of 66.6% [25], while a meta-analysis estimated a general prevalence of 31.1% in adults [37].

These findings are broadly consistent with previous reports describing changes in TMD-related symptoms following occlusal splint therapy; however, differences in study populations, diagnostic approaches, and outcome definitions limit direct comparisons. However, there is still debate in the literature about the optimal duration of the use of these devices. According to the protocol for the use of occlusal splints, there are no strict rules regarding the duration of use. Proposed mechanisms of occlusal splint therapy include alteration of occlusal contacts and reduction of parafunctional loading; however, the precise therapeutic mechanism remains uncertain [10-19].

Likewise, a greater reduction in joint symptoms was observed in individuals who recommended the use of the occlusal splint. Interestingly, a decrease in symptoms was also recorded in those participants who believed that the occlusal splint was not effective, suggesting that symptom reductions were also observed among participants who did not perceive the treatment as effective. Nevertheless, these findings should be interpreted cautiously given the subjective nature of some outcomes and the potential influence of patient expectations and therapeutic interaction.

The literature supports this interpretation: some studies suggest that occlusal splints may produce effects comparable to placebo, especially in subjective symptoms, and recent studies confirm that cognitive-emotional mechanisms associated with placebo generate clinical relief even in chronic pain. This suggests that part of the improvement could stem from contextual factors rather than the physical intervention itself [37,38].

Similarly, evidence from controlled studies suggests that improvements associated with occlusal splints may not always differ significantly from those observed in control or placebo groups [2-14]. These findings reinforce the importance of controlled studies to distinguish the specific effects of the intervention from contextual and nonspecific effects.

Finally, recognizing the potential influence of contextual factors and the natural course of TMD symptoms is important when interpreting changes observed during treatment and highlights the need for controlled studies to distinguish treatment-specific effects from nonspecific changes.

The present study has some limitations that must be considered when interpreting the results. First, this prospective, single-arm, uncontrolled before-after study limits the ability to establish causal inferences, and thus the observed changes should be interpreted as temporal associations rather than direct effects of the intervention. Participants were recruited from a specialized hospital-based clinical population, with many referred from other specialty services, which may have introduced selection bias and limits the generalizability of the findings to broader TMD populations. In the absence of a control group, regression to the mean and spontaneous fluctuations in TMD-related symptoms cannot be excluded as alternative explanations for part of the observed changes. Second, symptoms were assessed using a non-validated questionnaire, which may affect the internal validity of the measurements and limit comparability with other studies. Outcome assessment was not blinded, which may have introduced measurement or observer bias. A standardized TMD diagnostic protocol such as DC/TMD or RDC/TMD was not used, which may have limited the clinical characterization of specific TMD diagnoses, including myalgia, arthralgia, and disc displacement. Moreover, functional difficulty in mouth opening or closing was recorded as a patient-reported symptom, without standardized differentiation between pain-limited movement, mechanical locking, and other causes of functional limitation. No a priori sample size calculation was performed, and the relatively small sample sizes in some subgroup analyses may have limited the ability to detect differences between subgroups. Multiple symptom-specific comparisons were performed without adjustment for multiplicity, which may have increased the probability of type I error; therefore, these findings should be interpreted as exploratory. Potential confounding factors, including parafunctional habits, stress levels, adherence to splint use, and concomitant treatments or self-care measures outside the study protocol (e.g., analgesics, physiotherapy, or psychological interventions), were not systematically controlled or monitored. In particular, daily wearing time was not objectively monitored, and no predefined adherence threshold was established. Adverse events potentially associated with occlusal splint therapy were not systematically assessed or recorded; therefore, the safety of the intervention cannot be evaluated from the present study. Finally, no post-treatment follow-up was conducted after discontinuation of occlusal splint therapy; therefore, the persistence of the observed symptom changes beyond the treatment period could not be evaluated.

Finally, the inclusion of participants aged 14 to 60 years resulted in a heterogeneous age range encompassing adolescents and adults, who may differ in craniofacial development, hormonal status, psychosocial factors, and treatment response. This age heterogeneity may have influenced the observed changes and should be considered when interpreting the findings.

Future controlled studies should incorporate validated TMD assessment instruments, standardized diagnostic criteria, objective monitoring of splint adherence, systematic assessment of concomitant treatments and adverse events, prespecified outcomes with appropriate control of multiple comparisons, and longer post-treatment follow-up to determine the persistence of symptom changes.

## Conclusions

In this prospective, single-arm, uncontrolled before-after study, occlusal splint therapy was associated with reductions in self-reported TMD-related symptoms over the six-month treatment period, particularly joint fatigue, muscle pain, and headache. Given the absence of a comparator group, these findings should be interpreted as temporal associations and do not establish treatment efficacy or demonstrate prevention of disease progression. Further controlled studies with standardized diagnostic criteria and longer post-treatment follow-up are needed to confirm these findings.
